# Genome-Wide Exploration of miRNA Function in Mammalian Muscle Cell Differentiation

**DOI:** 10.1371/journal.pone.0071927

**Published:** 2013-08-21

**Authors:** Anna Polesskaya, Cindy Degerny, Guillaume Pinna, Yves Maury, Gueorgui Kratassiouk, Vincent Mouly, Nadya Morozova, Jeremie Kropp, Niels Frandsen, Annick Harel-Bellan

**Affiliations:** 1 Department Epigenetics and Cancer FRE 3377, Centre National de la Recherche Scientifique, Commissariat à l’Energie Atomique Saclay, Gif-sur-Yvette, France; 2 Université Paris-Sud, Gif-sur-Yvette, France; 3 Institut des cellules Souches pour le Traitement et l’Etude des Maladies monogéniques, Association Française contre les Myopathies, Evry, France; 4 Thérapie des maladies du muscle strié/Institut de Myologie, UM76– Université Pierre et Marie Curie Paris 6–Paris, France; 5 Exiqon A/S, Vedbaek, Denmark; Colorado State University, United States of America

## Abstract

MiRNAs impact on the control of cell fate by regulating gene expression at the post-transcriptional level. Here, using mammalian muscle differentiation as a model and a phenotypic loss-of-function screen, we explored the function of miRNAs at the genome-wide level. We found that the depletion of a high number of miRNAs (63) impacted on differentiation of human muscle precursors, underscoring the importance of this post-transcriptional mechanism of gene regulation. Interestingly, a comparison with miRNA expression profiles revealed that most of the hit miRNAs did not show any significant variations of expression during differentiation. These constitutively expressed miRNAs might be required for basic and/or essential cell function, or else might be regulated at the post-transcriptional level. MiRNA inhibition yielded a variety of phenotypes, reflecting the widespread miRNA involvement in differentiation. Using a functional screen (the STarS - Suppressor Target Screen – approach, i. e. concomitant knockdown of miRNAs and of candidate target proteins), we discovered miRNA protein targets that are previously uncharacterized controllers of muscle-cell terminal differentiation. Our results provide a strategy for functional annotation of the human miRnome.

## Introduction

The microRNA (miRNA) machinery [1] has an essential function during development [2]. MicroRNAs (miRNAs) [1] are encoded in intergenic or intronic sequences as long precursors that are sequentially processed by the endonucleases Drosha and Dicer into short double-stranded sequences [3]. They regulate gene expression at the post-transcriptional level: in the cytoplasm, they guide the RISC complex, an Argonaute-containing complex of proteins, toward a target messenger RNA. The RISC complex cleaves the target messenger, or else inhibits its translation and/or accelerates its decay [4]. MiRNAs control the balance between cell proliferation, cell differentiation and cell death [3]. However, a limited number of miRNAs, have been studied at the functional level in specific differentiation programs, and genome-wide phenotypic analyses are scarce or absent in mammalian systems. Thus, a few miRNAs, miR-133, miR-1, miR-206, miR-181 and miR-27a, have previously been shown to be important in skeletal muscle cell terminal differentiation [5]. Here, we implemented a genome-wide miRNA loss-of-function screen using LNA-modified synthetic antisense oligonucleotides [6] in LHCN, a human skeletal muscle precursor cell line. The depletion of 63 miRNAs impacted on LHCN differentiation. Moreover, using a phenotypic screen based on co-suppression of miRNAs and putative targets that we named the STarS assay, we identified important proteins whose role in controlling differentiation had not been previously identified.

## Results

### The Depletion of 63 miRNAs Impacts on Skeletal Muscle Cell Terminal Differentiation

**Figure 1:**
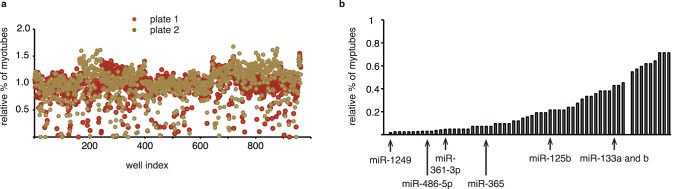
Screening a genome-wide LNA antisense library. (a) Plate-well series plot of the screen: library LNAs (100 nM) were transfected into LHCN and differentiation was monitored 7 days later (relative percentage of myotubes standardized on the mock-transfected control) (b) Proportion of myotubes for hits with inhibited differentiation (relative percentage of myotubes standardized on the control); notable hits are marked with an arrow (previously characterized miRNAs, or miRNAs selected for further analysis).

A library of LNA antisense inhibitors targeting 870 miRNAs (listed in miRBase v12) was screened on a differentiating human muscle precursor cell line, LHCN [7], by transfecting individual inhibitors (in duplicate) prior to inducing differentiation. Muscle cell terminal differentiation involves the fusion of myoblastic precursor cells into large multinucleated post-mitotic cells (myotubes) that express muscle-specific markers such as muscle Myosin Heavy Chain (MHC) and Muscle Creatine Kinase (MCK). Differentiation was monitored on an Array Scan VTI automated microscope. Cells were reconstructed and nuclear and cytoplasmic Regions of Interest (ROI) were identified using the vHCS scan software (Figure S1a). The proportion of multinucleated MHC-positive myotubes (i. e., cells that are positive for MHC fluorescence and have at least 3 nuclei) was scored in each individual sample and control well (raw data in File S1: SI1_LNA Screen 1 raw data.xls). A plate-well series plot of the results is shown in Figure 1a. Candidate miRNAs with differences to the negative control ≥2 SDs (corresponding to a proportion of myotubes smaller or equal to 39% of the control) were selected using the Spotfire Decision Software and retested in a secondary screen (in triplicates): we monitored, in addition to the percent of multinucleated myotubes the total numbers of nuclei. Thresholds of 27% and 25.2%, corresponding to deviations of ≥2 SDs from the negative controls in the secondary screen, were set for inhibition of differentiation or decreased cell number phenotypes respectively. However, this threshold was not applied for increased differentiation or increased cell growth, as the conditions of the experiment were optimized to monitor a decrease but not an increase of these parameters (full confluence, low serum etc.). A proportion of candidates (63%) were confirmed in the secondary screen (Table I), i. e., a total of 63 hits. Among the hits were the miR-133 family members previously characterized as important muscle-specific miRNAs [8], which support the robustness of our screen. Moreover, as an additional quality control test, 18 hits were re-tested using new batches of LNAs: all hits were confirmed, further increasing the confidence in the screen results (Figure S1b). Note, however, that one out of eighteen hits, miR-18b, was not detected by RT-QPCR in our cells and might be a false positive. Finally, whenever monitored, miRNA knockdown was observed, at levels of inhibition ranging from 70 to 99% and with a high degree of specificity (Figure S1c and d).

**Table 1:** List of hits.

Hits of functional screen	Relative percentage of myotubes1, % of control	p value, Mann Whitney^2^	Relative cell count3, % of control	p value, Mann Whitney
**Inhibited differentiation**				
hsa-mir-100	56.13	***	97.25	*
hsa-mir-106a/hsa-mir-17	69.91	***	93.99	***
hsa-mir-1227	7.12	***	91.11	*
hsa-mir-1233	11.88	***	73.07	***
hsa-mir-125b	21.37	***	80.27	***
hsa-mir-1267	2.37	***	75.12	***
hsa-mir-130b*	27.31	***	86.11	***
hsa-mir-138-1*	57.01	***	80.1	***
hsa-mir-145	23.75	***	83.88	***
hsa-mir-1538	19	***	85.59	***
hsa-mir-18b*	15.44	***	75.64	***
hsa-mir-223	30.88	***	92.11	-
hsa-mir-296-5p	23.75	***	93.31	**
hsa-mir-326	14.25	***	94.34	***
hsa-mir-331-3p	4.75	***	90.22	***
hsa-mir-339-5p	66.39	***	92.79	**
hsa-mir-365	7.13	***	77.53	***
hsa-mir-429	54.63	***	85.25	***
hsa-mir-454	33.25	***	87.31	-
hsa-mir-455-3p	42.76	***	96.4	-
hsa-mir-484	4.83	***	78.73	-
hsa-mir-485-3p	4.75	***	71.49	***
hsa-mir-501-3p	69.25	***	91.25	***
hsa-mir-512-5p	21.37	***	72.89	***
hsa-mir-532-3p	9.5	***	85.93	***
hsa-mir-541	69.87	***	97.77	-
hsa-mir-600	35.63	***	93.48	-
hsa-mir-625*	28.5	***	72.89	***
**Hits of functional screen**	**Relative percentage of myotubes** 1, **% of control**	**p value, Mann** **Whitney test**	**Relative cell count** 2, **% of control**	**p value, Mann Whitney test**
hsa-mir-636	2.37	***	81.98	***
hsa-mir-663	21.38	***	84.73	***
hsa-mir-664	7.13	***	82.85	***
hsa-mir-766	45.13	***	73.24	***
hsa-mir-770-5p	19	***	75.47	***
hsa-mir-93*	9.5	***	92.1	-
**Inhibited differentiation & low cell count**			***	
hsa-let-7b*	4.75	***	28.64	***
hsa-mir-1224-3p	2.38	***	51.46	***
hsa-mir-1228	2.38	**	9.43	***
hsa-mir-1249	1.66	***	53.17	***
hsa-mir-125a-5p	19	***	69.8	***
hsa-mir-1260	7.12	***	61.75	***
hsa-mir-1280	11.88	***	68.95	***
hsa-mir-129-3p	9.5	***	65.64	-
hsa-mir-1296	9.5	***	36.36	***
hsa-mir-133a/hsa-mir-133b	42.75	*	0.85	***
hsa-mir-150	4.75	***	60.37	***
hsa-mir-197	4.75	***	27.79	***
hsa-mir-204	2.85	***	27.44	***
hsa-mir-328	0.1	**	30.87	***
hsa-mir-342-3p	33.25	***	58.83	***
hsa-mir-346	7.13	***	69.13	***
hsa-mir-361-3p	4.51	***	9.6	***
hsa-mir-483-3p	3.56	***	68.61	***
hsa-mir-486-5p	2.85	***	34.48	***
hsa-mir-574-3p	2.61	***	43.22	***
hsa-mir-629*	16.62	***	67.23	***
hsa-mir-885-5p	4.75	***	43.73	***
**Inhibited differentiation & high cell count**				
hsa-mir-193b	38.04	***	102.74	*
**Hits of functional screen**	**Relative percentage of myotubes** 1, **% of control**	**p value, Mann Whitney test**	**Relative cell count** 2, **% of control**	**p value, Mann Whitney test**
hsa-mir-369-3p	61.75	***	103.6	*
hsa-mir-381	61.75	***	105.31	*
hsa-mir-886-5p	38.04	***	112.86	***
hsa-mir-940	21.37	***	112.35	***
**Enhanced differentiation**				
hsa-mir-98	104.51	*	87.82	***
**High cell count**				
hsa-mir-631	92.63	**	103.43	***

1 see Material and Methods;

2 *: p<0.05; **: p<0.01; *** p<0.005; − = not significant.

3 total number of nuclei.

LNA inhibitors induced various phenotypes (summarized in Table I). Inhibition of differentiation was observed in most instances. In the vast majority of the corresponding hits, inhibition was greater than 50% (Figure 1b). However, in some cases, and despite the fact that the assay was designed to detect inhibition, we instead observed activation of differentiation (Table I). LNAs affected various parameters: the proportion of differentiated cells (myotubes), the size of myotubes or both. Interestingly, some LNAs also impacted on the total cell number, in either a positive or a negative manner. In muscle, as well as in many other tissues, differentiation and proliferation are mutually exclusive, and the balance between the two processes is strictly regulated. Hit miRNAs affecting cell numbers might be expected to be involved in the control of this essential balance, and their function might not be restricted to muscle. Thus a subset of hit miRNAs could affect differentiation in an indirect manner, by impacting on cell proliferation and/or cell survival. In this category, miR-361-3p inhibition dramatically decreased the number of cells, suggesting that this miRNA controls pathways involved in cell survival. MiR-940 inhibition, on the other hand, consistently resulted in a 10% higher number of cells than in the control populations. Given that cells were seeded at full confluence for differentiation, a higher increase could not be expected. We thus believe that this 10% increase might be “biologically” significant, and implies that this miRNA might be required for proper growth control. Finally, we also identified miRNAs that are negative regulators of differentiation, since one or more of the differentiation parameters was enhanced when they were inhibited. Quite unexpectedly, miR-98, a member of the let-7 family previously characterized as pro-differentiation molecules [9], was detected as anti-differentiation in our cell model.

MiR-1 and miR-206 were not detected in our screen, in contrast to previously published results [8].This might be due either to the high homology and possible redundancy between these two miRNAs or to the experimental model used here. Finally, miR-27a, a miRNA involved in embryonic muscle differentiation [10] was not detected in our screen either, most likely because it is only involved in very early steps of differentiation, whereas our screen addressed later steps in the differentiation process. Note, in any case, that there is usually a certain rate of “false negative” in global screens.

### Phenotypic Importance does not Strictly Correlate with Differential Expression

**Figure 2:**
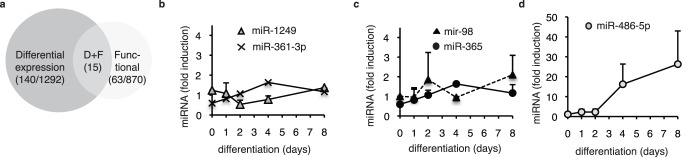
Lack of correlation between differential expression and functional significance. (**a**) Comparison between expression and functional results: distribution of miRNAs with differential expression (ratio between proliferation and differentiation ≥2, dark grey), with functional impact (light grey) or with both. (**b–d**) Time course of expression for miR-361-3p, miR-1249, miR-365, miR-98 and miR-486-5p during terminal differentiation. LHCN cells were placed under differentiation conditions, and RNAs were extracted at indicated times and analyzed by RT-QPCR (mean of biological duplicates); results are shown as fold inductions as compared to proliferating cells.

MiRNA expression profiles were established for 97% of the miRNAs tested in the functional screen (see File S2: SI2_Comparison of miRNAs tested in the two screens.xls), using arrayed RT-QPCR (see File S3: SI3_Expression screen.xls, for the raw data; accession number: GSE45957). A comparison between the list of miRNAs showing differential expression during terminal differentiation and the list of miRNAs having a functional impact showed that the majority of the functionally important miRNAs hits were not scored as being differentially expressed (see Figure 2a). Indeed, even though the number of miRNAs that were up- or downregulated during differentiation was unexpectedly high, a minority of the hits (15 miRNAs, Figure S1e) was found to be both differentially expressed and functionally important for myoblast differentiation. Moreover, the majority of the differentially expressed hits were not dramatically induced or dramatically repressed. This observation was further confirmed for a selection of hits analyzed individually (Figure 2 b, c) that showed little or no modification of expression during differentiation. We have selected, for individual analysis, hit miRNAs linked to strong phenotypes in the original loss-of-function screen and that had various expression profiles during terminal differentiation. This selection of hits included two pro-differentiation miRNAs, miR-1249 and miR-365; one anti-differentiation miRNA, miR-98; and a miRNA required for myoblast survival, miR-361-3p – note that miR-98 and miR-365 have similar expression profiles but have opposite effects on differentiation. We also analyzed a miRNA strongly induced upon differentiation, miR-486-5p (Figure 2d), a pro-differentiation miRNA in our cell model and recently linked to cardiac cell differentiation [11].

### Validation of Selected Hits by Mimics

**Figure 3:**
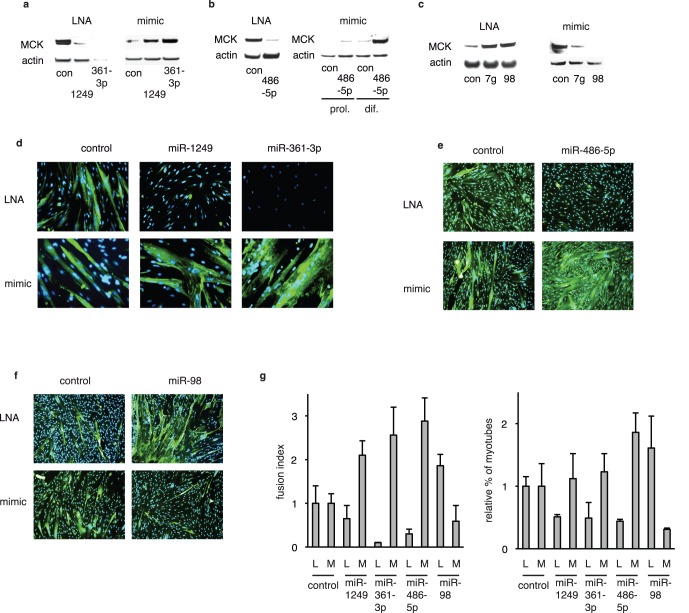
Validation of selected hits by gain-of-function assays. LHCN cells were transfected with indicated LNAs (75 nM) or mimics (10 nM for miR-486-5p and 25 nM for all others; con: irrelevant control sequence) and placed under differentiation conditions, except when indicated (prol. = proliferation conditions); extracts were analyzed by western blots (**a–c) -** this experiment was reproduced 3 times - or by immunostaining (**d–f)**, green: MHC, blue: Hoechst; (typical experiment that was run in triplicate and reproduced twice) at 7 days of differentiation; (**g)** statistical analysis of the results shown above: images were acquired and analyzed on an Operetta (Perkin Elmer); shown are the relative fusion index (see Material and Methods) and the relative percentage of MHC positive cells, with controls set to 1; L: LNA antisense, M: mimic.

Gain-of-function assays using mimics were used to further explore the selected miRNAs functionally. A high proportion (5/6 = 80%) of mimics affected differentiation in the expected manner (Figure 3): ectopic expression of miR-486-5p, miR-1249 and miR-361-3p resulted in increased expression of the differentiation marker MCK, whereas inhibition of these miRNAs reduced MCK expression (Figure 3a, b); conversely, ectopic expression of let-7 miRNAs (miR-98 and another member of the family, let-7g) decreased MCK expression, whereas inhibition of these miRNAs increased MCK expression (Figure 3c). Most interestingly, miR-486-5p was able to induce differentiation under proliferation conditions (Figure 3b), implying that ectopic expression of this miRNA overrides the extremely stringent control of proliferation and differentiation operating in myoblast cells. The other mimics tested under proliferation conditions did not induce any phenotypic changes in myoblasts (data not shown). Under differentiation conditions, ectopic miR-1249, miR-361-3p and miR-486-5p all induced precocious differentiation, and inhibition of let-7 miRNAs had the same effect (Figure S2a). Although miR-1249, miR-361-3p and miR-486-5p mimics all impacted on differentiation in a similar manner, detailed analysis of the phenotypes of treated cells showed subtle differences in the morphology of differentiated myotubes (Figure 3d, e, g). Thus, miR-1249 and miR-361-3p mimics increased the fusion index (proportion of nuclei in myotubes) as compared to controls, whereas miR-486-5p mimic increased both the percentage of myotubes and the fusion index (Figure 3 e, g). Only one mimic out of six, miR-365, had no effect on differentiation (Figure S2b). The reason for this negative result is unclear. Mir-365 appears to be highly expressed in LHCN cells (as judged from RT-QPCR results, Figure S2c) and might thus be in excess in the cells.

### Identification of Essential miRNA Targets Using the STarS Assay

**Figure 4:**
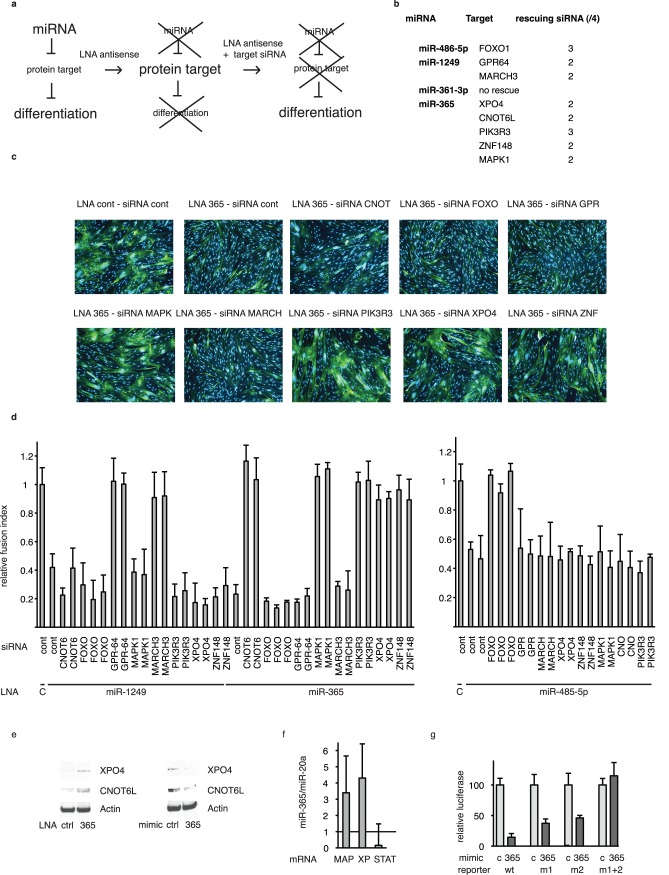
STarS, a genetic suppressor assay to identify phenotypically important miRNA targets. (**a**) Rationale of the assay. (**b**) Unbiased identification of phenotypically important targets of miR-486-5p, miR-1249 miR-361-3p and miR-365. MiRNAs were knocked down using LNA inhibitors (50 nM), and targets were knocked down using siRNAs (50 nM); differentiation was monitored by quantifying the fusion index on day 7; identified targets and number of rescuing siRNAs are listed in (ERK: ERK1, XPO:XPO4, MAPK: MAPK1). (**c**) Fluorescence images of LHCN cells transfected with miR-365 LNA along with indicated siRNAs, and analyzed at day 7 by immunofluorescence after staining with anti-MHC antibodies (green) and counterstaining with DAPI (blue); cont: control; CNOT: CNOT6L; FOXO: FOXO1; GPR: GPR-64; MAPK: MAPK1; MARCH: MARCH-3; ZNF; ZNF-148. (d) Specificity of the STarS assay. LHCN cells were transfected as above with mixtures of LNAs and siRNAs (2–3 different siRNAs per gene), as indicated, and analyzed 7 days later by quantifying the fusion index; shown are relative values, using cells transfected with control siRNA and control LNA as a reference. (**e**) CNOT6L and XPO4 proteins are regulated by miR-365. LHCN cells were treated with miR-365 LNA or mimic as indicated, and CNOT6L and XPO4 proteins were detected by western blotting 48 h later. (**f**) XPO4 and MAPK1 are direct targets of miR-365: analysis by TAP-Tar. C2C12 mouse myoblast cells stably transduced with a vector expressing a tagged version of AGO2 were transfected with biotinylated miR-365, or miR-20a as a control, and extracts were submitted to tandem affinity purification followed by detection of associated mRNAs by RT-QPCR (MAP: MAPK1; XP: XPO4; STAT: STAT3). Values are means of 3 independent biological replicates, with duplicate measurements, of the ratios between values for miR-365 and values for miR-20a. (**g**) CNOT6L is a direct target of miR-365, analysis by a standard Renilla luciferase reporter assay, using 3.230 kb of CNOT6L 3′UTR, either wild type (wt) or with the predicted binding sites for miR-365 mutated individually (M1 orM2) or in combination (M1+2), transfected into C2C12 cells along with miRNA365 precursor (365) or a control precursor (C); shown are the ratio between the control precursor (set to 100%) and miR-365 precursor, after standardization on Firefly luciferase; results of a typical experiment with triplicate measurements that was reproduced 3 times.

MiRNAs impact on cell biology by downregulating specific gene targets. In order to explore how our selection of hit miRNAs impact on skeletal muscle cell differentiation, we undertook the task of identifying functionally important gene targets. In mammals, unbiased target identification can be performed by Affymetrix mRNA profiling [12] or biochemical pull-downs [13], [14]. However, these assays do not document the functional importance of identified targets. In order to directly address this question, we set up an assay similar to a standard genetic suppressor screen, based on the following rationale (Figure 4a): the phenotype observed upon miRNA downregulation by LNA inhibitors is, at least to a significant extent, due to overexpression of functionally important target proteins; consequently, concomitant down-regulation of a miRNA and of important target proteins should result in partial if not complete correction of the phenotype. We previously used this assay to characterize Hox-A11 as an important target of the pro-differentiation miRNA miR-181 in myoblasts [15]. As an additional proof of principle, in the present study, we used the same approach to demonstrate that Lin-28 is a functionally important target of the anti-differentiation miRNA let-7. Let-7 has previously been shown to control the expression of HMGA2 [16], Lin-28 [17] and IMP-1 [18] proteins, and, in keeping with these observations, these proteins were upregulated upon let-7 inhibition in our human myoblast cell line (Figure S3a). Whereas inhibiting let-7 increased differentiation, downregulating Lin-28 at the same time decreased differentiation back to normal levels, as monitored by the expression of the muscle specific marker MCK (Figure S3b) and as judged by the size of myotubes (Figure S3c). These results confirm that Lin-28, an activator of differentiation in muscle cells [17], [19], is a functionally important target of let-7. As expected, other let-7 targets (HMGA2 and IMP proteins) were not affected by Lin-28 knockdown (Figure S3b) whereas IGF-2, a target of Lin-28 [19], was downregulated. These results validate this stringent assay, which is thus usable for unbiased screens of miRNA targets, designed to directly identify phenotypically important targets. We named this assay STarS (Suppressor Target Screen).

As a first proof-of-principle, we restricted our screen to targets predicted by 2 algorithms (www.targetscan.org and http://genie.weizmann.ac.il/pubs/mir07/mir07_prediction.html) for four hit miRNAs: miR-486-5p, miR-361, miR-365 and miR-1249 (list of the targets tested in Figure S3d). The experimental setting was similar to that used in the miRNA inhibitor screen, except that cells received, instead of the LNA alone, a mixture of LNA miRNA inhibitors along with siRNAs downregulating miRNA putative targets prior to being placed under differentiation conditions. In a first screen, two siRNAs were tested for each potential target, and hits (for which at least one siRNA induced recovery of the phenotype as monitored by the proportion of myotubes) were screened again with 2 additional siRNAs per gene (see File S4: SI4_Raw data STarS.xls). Potential targets were considered to be hits when at least 2 siRNAs caused significant levels of phenotypic rescue (Figure 4b for a summary of the results). For example, FOXO1 was identified as a phenotypically important target of miR-486-5p (Figure 4b), in keeping with previous observations in cardiac cells [11], showing the importance of this target in both skeletal and cardiac muscle as well as validating the present screen. The specificity of phenotypic rescue was first confirmed for miR-365, using siRNAs against putative targets of this and other miRNAs, by monitoring differentiation markers using immunofluorescence (Figure 4c) and western blot (Figure S3e). Subsequently, targets identified for any given miRNA were systematically tested upon downregulation of other miRNAs (Figure 4 d). In all cases, downregulating the targets of a given miRNA rescued differentiation upon inhibition of this miRNA, but did not do so when a different miRNA was inhibited: for example, downregulating miR-365 targets (PIK3R3, MAPK1, XPO4 and CNOT6L) rescued inhibition by miR-365 LNA but not by miR-1249 or miR-486-5p LNAs. These data demonstrate the specificity of the STarS assay and indicate that the identified targets are indeed in the same pathway as the miRNA inhibited in the assay and under its control, albeit possibly as indirect targets. The STarS assay demonstrates the phenotypic importance of protein targets, but does not demonstrate that they are directly regulated by miRNAs. We addressed this issue for some target proteins, and MAPK. CNOT6L, XPO4 were regulated by miR-365 overexpression or downregulation (Figure 4e), and could be validated as direct targets, either by a TAP-Tar assay [14] in which mRNA targets are biochemically pulled down (Figure 4f) or, when this assay was not sensitive enough, by a standard reporter assay (Figure 4g).

## Discussion

MiRNAs contribute to gene regulation only at the post-transcriptional level, and they are often considered as “fine tuners”. Moreover, miRNAs can function redundantly; and, in many cases, several miRNAs co-regulate the same target gene. Consequently, individual miRNA molecules are often expected to be non-essential molecules. However, our genome-wide functional screen of miRNAs involved in mammalian muscle cell differentiation identified 63 miRNAs that are required for normal differentiation. Note that it is possible that important miRNAs are ignored in our functional screen (miR-1 and miR-206, for example were not detected), in particular due to miRNA redundancy. Indeed, miRNA inhibitors were designed to inhibit individual members of miRNA families, and, for example, miR-181, a miRNA essential for differentiation [15] was below the threshold of detection in this screen, likely due to the high redundancy of the miR-181 family. The high number of hits was quite unexpected as compared to the few miRNAs that were found to be essential in a previously published screen implemented in *C. elegans* [20] (in which, however, only 10% of the genome miRNAs were analyzed). Our result underscores the importance of “fine-tuning” by miRNAs.

Quite unexpectedly, the 63 hits were not systematically differentially expressed during differentiation, and the proportion of miRNAs that were differentially expressed in the hit population was rather low (about 25%). Some of the constitutive miRNAs might be involved in biological essential functions and might impact on differentiation in an indirect manner. But, for some of these miRNAs that are directly involved in differentiation, this result suggests that miRNA activity is modified upon differentiation. MiRNA activity might be regulated through modulation of miRNA targets, for example via the modification of miRNA binding sites in the target 3′UTRs. MiRNA activity might also be regulated by RNA binding proteins that control mRNA translation, such as IGF2BPs or CPEB proteins. In any case, the activity of individual miRNA molecules cannot be directly inferred from their expression profiles, underscoring the importance of functional screens, based on the direct estimation of the influence of miRNA depletion on cell phenotypes, to decipher miRNA function.

The phenotypes observed were diverse reflecting the involvement of miRNAs in multiple steps of the differentiation process. Using the STarS assay, we were able to identify functionally important targets of hit miRNAs. Some of these hit target proteins have been previously characterized as inhibitors of myotube differentiation: the miR-486-5p target FOXO1 [21] and the miR-365 target ERK2 [22]. Others, in contrast, have never been linked to skeletal muscle differentiation. The miR-365 target ZNF148 (ZBP-89) is a zinc finger protein that was shown to regulate multiple promoters in myoblast cells [23]. The miR-365 target PIK3R3 binds the IGFR1 receptor and is involved in regulating cell proliferation by the IGF cytokines [24]. CNOT6L is a subunit of the CCR4 complex [25], which controls gene expression at multiple levels, including shortening of mRNA polyA tails in the cytoplasm, and a repressor of p27, a proliferation inhibitor required for differentiation [26]. Exportin-4 (XPO4) is a nuclear export protein that inhibits EIF5A2 [27], a translation regulator that seems to be important for skeletal muscle cell differentiation [28].

Downregulating miRNA targets did not systematically fully reconstitute differentiation. In particular, downregulating the miR-365 target CNOT6L restored cell fusion and MCK expression, but not MHC expression (Figure 4c et S3e). In contrast, downregulating XPO4 (or PIK3R3), which are also targets of miR-365, restored all measured parameters. These data indicate that distinct pathways, leading to MHC expression, cell fusion and/or MCK expression are all under the control of miR-365.

The miR-1249 target GPR64 (He6) is a G-protein–coupled orphan receptor preferentially expressed in epididymis [29], and its connection with skeletal muscle is quite unexpected. However, GPR64 is also a target of the pro-differentiation transcription factor RUNX2 in osteoblasts [30], a differentiation pathway mutually exclusive with myoblastic differentiation for mesodermic precursor cells. Finally, the miR-1249 target MARCH3 is an unexplored membrane RING protein belonging to the E3-Ubiquitin ligase family.

In summary, we describe here a genome-wide screen for miRNA function in skeletal muscle cells. We show that the depletion of an unexpectedly high number of miRNAs (63) impacts on normal progression of terminal differentiation. We undertook the analysis of some of the pathways controlled by 4 hit miRNAs, using an assay that we called the STarS assay, a genetic suppressor screen that directly identifies phenotypically important targets. Our data revealed the unsuspected importance of several proteins, either not previously linked to muscle differentiation or else of completely unknown function, thus opening up new avenues of research. Thus, our study provides a convenient strategy that can facilitate the functional annotation of the human miRnome.

## Materials and Methods

### Cell Culture and Transfections

LHCN-M2 immortalized human myoblasts [7] were cultured under standard conditions. Briefly, cells were grown in 80% DMEM (Invitrogen 61965), 20%199 (Invitrogen 41150) supplemented with 20% FBS (PAA A 04306-0360, lot 731), penicillin/streptomycin (Invitrogen), plasmocin 2.5 µg/ml final (Invivogen). For transfection and differentiation assays, cells were seeded on collagen-coated plates (50 mg of rat tail collagen - Sigma C 7661 - in 500 ml of 0.2% acetic acid in ddH_2_O) at 2×10^4^ cells/well of 96-well plates, or 5×10^5^ cells/well of 6-well plates. Differentiation was induced by switching to DMEM/antibiotics supplemented with 0.01 mg/ml insulin and 0.1 mg/ml transferrin (Invitrogen). The library of microRNA inhibitors with normalized Tm (miRCURY LNA microRNA inhibitor library v. 12) was provided by Exiqon (Vedbaek, Denmark). The library contains 918 inhibitors targeting 870 human microRNAs listed in miRBase v. 17.0. The positive control was a miR-181 LNA inhibiting the whole miR-181family (Exiqon) and the negative controls were a mutant of this oligonucleotide ([15] or mock transfected cells. In addition, an “All Stars Death” siRNA mix (Qiagen) was also run to monitor transfection efficiency. SiRNAs were obtained from Qiagen (Target accession numbers are listed in Figure S3 and sequences are available upon request). A control siRNA was used in all experiments (uagcaaugacgaaugcgua). LNAs, mimics or siRNAs were transfected by reverse transfection using LipoRNAiMAX (Invitrogen) in 96-well plates using a pipetting robot (Microlab Star, Hamilton).

### MicroRNA Profiling

Arrayed RT-QPCR profiling was performed by Exiqon. For individual RT-QPCR, total RNA was isolated with TRI Reagent Solution (Ambion), and assayed using the miRCURY LNA Universal RT microRNA PCR system (Exiqon), or else with the TaqMan MicroRNA Assay Kit. MiR16, constitutively expressed, was used as an internal standard.

### Image Acquisition and Analysis

Terminal differentiation was monitored using an Array Scan VTI (Cellomics) or an Operetta (Perkin Elmer) High Content Screening system. These automated microscopes allow the systematic acquisition and analysis of epifluorescence images in 96-well plates. We used the colocalization bioapplication of the vHCS scan software (Cellomics), and the Harmony Image analysis software (Perkin Elmer) to analyze images acquired in two channels with a 5× (Cellomics) or a 10× (Perkin Elmer) objective in high-resolution camera mode. Nine (5×) or eleven (10×) pictures were scanned per well (covering approximately 50% of the well surface) in 96-well plates. On both systems, the first channel was used to automatically focus on the cell preparation based on the Hoechst signal (Hoechst Channel) and then to identify and quantify nuclei, while the second was used for myotube identification, based on the MHC labeling (FITC channel). Applying intensity thresholds, nuclei and cell/myotubes were segmented to define 2 corresponding Regions Of Interest (ROI). Four parameters were analyzed: the percentage of myotubes (i.e. the percentage of cell ROI expressing MHC and with more than 3 nuclei), the fusion index (i. e. the number of nuclei in myotubes containing at least three nuclei above the total number of nucleai), the size of myotubes (i.e. the area of MHC-positive cell ROIs) and the cell count (total number of nuclei). In some cases, parameters were standardized on the Mock transfected control. The percentage of multinucleated myotubes was used to select hits in the primary screen and the residual viability (nuclei count per valid acquisition field) was added as an exclusion parameter in the secondary screen.

### Data Analysis

For the purpose of the primary screen, inhibitor miRNAs were selected as candidates when their activity values were found below 2 standard deviations (SD) from the negative control (corresponding to an approximate confidence interval of 95%). In the secondary screen, the Mann-Whitney test was used as a significance test. The significance threshold was set at 0.05 for the purpose of the selection of hit miRNAs.

### Antibodies

Western blotting and immunofluorescence were performed using standard procedures. Antibodies were: MHC (Sigma My-32), MCK (Santa Cruz sc-15161), Lin-28 (R&D AF3757), HMGA2 (R&D AF3184), IMP-2 (Abnova H00010644-A01), IMP-1 and 3 (kind gift of Pr. F. C. Nielsen), IGF-2 (R&D AF792), nucleolin (Sigma N2662), beta-actin (Sigma A5441), HRP-conjugated secondary antibodies (Sigma), Alexa Fluor-488-conjugated anti-mouse secondary antibody (Invitrogen A11029). Hoechst 33258 was used for staining of nuclei.

### TAP-Tar

C2C12 mouse myoblastic cells stably transduced with a vector expressing a tagged version of AGO2, or with the empty vehicle vector for the control, were transfected with biotinylated miR-365, or miR-20a as a control. Extracts were submitted to tandem affinity purification, first with anti-Flag antibodies (to immunoprecipitate AGO complexes) and then with streptavidin (to purify miR-365 complexes). Target mRNAs were analyzed by RT-QPCR.

### Reporter Assays and Mutagenesis

A pSiCheck reporter vector (Promega) containing 3.230 kb of either CNOT6L 3′UTR wild-type (nt 2710 to 5940) or mutant (the miR-365 seeds, gggcatt, were mutated into gtgcagt using the AccuPrime Pfx site-directed mutagenesis system - Life Technology) was transfected into C2C12 cells (a murine myoblast cell line), along with a control miRNA precursor or miR-365 precursor (50 nM final concentration). Luciferase was measured 48 days later, in triplicates.

## Supporting Information

Figure S1 a: Myotube reconstruction using mock transfected cells; mock transfected LHCN cells were differentiated for 7 days and stained with DAPI and anti-MHC antibodies; stained cells were analyzed on the Array Scan VTI microscope and reconsructed; green: MHC, blue: Hoechst. b: Confirmatory screen for 18 selected targets with new batches of LNA. LHCN cells were transfected with indicated LNAs and analyzed after 7 days of differentiation, following staining with DAPI and anti-MHC antibodies. Four parameters were measured as indicated. The background given by the “all death” siRNA was subtracted. c: MiRNA Knockdown. RNAs were extracted from cells treated with indicated LNAs for the indicated period of times, and the target miRNA was quantified by RT-qPCR. Values are relative to control samples, i. e. cells that received a control, scrambled LNA (set to 1), after standardization on miR-16 (a constitutive miRNA) content, and are means of 2–3 independent experiments with duplicate measurements (s.d.) d: MiRNA knockdown is specific. RNAs were extracted from cells treated with miR-98 or let-7g LNA for 2 days, and indicated miRNAs were quantified by RT-qPCR. Values are relative to control samples, i. e. samples that received a control, scrambled LNA (set to 1), after standardization on miR-16 content of the samples, and are means of 3 independent experiments with duplicate measurements (s. d.). Let-7 family LNAs are specific of the let-7 family (there is no significant inhibition of miR-365), and also more efficient on the targeted isoforms, even though each LNA also inhibits the other isoform. e: List of miRNAs that are hits in the phenotypic screen and differentially expressed.
(EPS)

Figure S2 a: early differentiation of cells treated with miR-1249, miR-361-3p, miR-486-5p mimics or let-7 LNA antisenses. LHCN cells were transfected with mimics or LNAs as indicated and analyzed after 4 days of differentiation by western blot (upper panel) or following staining with DAPI and anti-MHC antibodies (lower panels). At day 4, control cells do not show any sign of differentiation, whereas treated cells express MHC and form myotubes; con = control. b: MiR-365 mimic does not impact on differentiation. LHCN cells were transfected with mimics or LNAs as indicated and analyzed after 7 days of differentiation by western blot (upper panel) or following staining with DAPI and anti-MHC antibodies (lower panel). ctrl: control; 365: miR-365. c: RT-Q-PCR analysis of miRNA expression in LHCN at day 0 of differentiation; shown are the Cts; miR-16 was used as a reference.
(EPS)

Figure S3 a: Upregulation of Let-7 protein targets. LHCN cells were transfected with let-7g or miR-98 LNA antisenses as indicated, and expression of the indicated proteins was monitored by western blot at indicated times. Nucleolin is an example of unmodified protein. b: Lin-28 is a target of Let-7. LHCN cells were transfected with miR-98 or control LNA antisenses at indicated doses, along with control or Lin28 siRNAs at indicated doses. Differentiation was monitored by western blot analysis of the muscle marker MCK; downregulating Lin-28 restored normal levels of MCK, as well as of IGF-2, but did not affect other Let-7 targets (HMGA-2; IMP-2). c: Lin-28 siRNA restores normal differentiation increased by let-7 LNAs. LHCN cells were transfected with indicated LNAs and siRNAs, and analyzed at day 7 by immunofluorescence after staining with anti-MHC antibodies and counterstaining with DAPI (upper panel); the fusion index was quantified (lower panel); ctrl: control. d: List of targets tested using the STarS assay in Figure 4d. e: Monitoring differention by MCK expression in the experiment described in Figure 4c. LHCN cells were transfected with miR-365 LNA along with indicated siRNAs (2 siRNAs per gene), and extracts were analyzed at day 7 by western blot with indicated antibodies; cont: control; CNO: CNOT6L; PI3: PIK3R3 MAP: MAPK1; XPO: XPO4.
(EPS)

File S1 **LNA screen raw data.**
(XLS)

File S2 **Lists of miRNAs tested in the LNA antisense screen and in the expression screen.**
(XLS)

File S3 **Raw data of expression screen.**
(XLS)

File S4 **Raw data of the STarS assay.**
(XLS)
